# The linkage between opioid treatment programs and recovery community centers: results of a survey of OTP directors

**DOI:** 10.3389/fpubh.2025.1532374

**Published:** 2025-08-07

**Authors:** Bettina B. Hoeppner, Alivia C. Williamson, Cora Nicoll, Diadora Finley-Abboud, Allison Futter, Judeline Joseph, Angela Burton, Drew Hickman, Anita Bradley, Felecia Pullen, Andre Johnson, Susanne S. Hoeppner

**Affiliations:** ^1^Department of Psychiatry, Massachusetts General Hospital, Boston, MA, United States; ^2^Detroit Recovery Project, Detroit, MI, United States; ^3^Northern Ohio Recovery Association, Cleveland, OH, United States; ^4^The PILLARS, New York, NY, United States

**Keywords:** recovery community centers, peer recovery support services, substance use disorder, recovery, addiction, definition of recovery

## Abstract

**Objective:**

Medications for opioid use disorder (MOUDs) are regarded as the gold-standard treatment for opioid use disorder in the United States and are widely used in other countries. In the US, the country most impacted by the opioid epidemic, opioid treatment programs (OTPs) are the primary avenue of accessing MOUDs. US federal guidance states that treatment providers should connect patients with recovery community centers (RCCs), if available. RCCs have emerged relatively recently. It is not clear to what extent OTP directors are aware of RCCs. Close collaboration is needed especially in Black communities, as Black Americans face significant disparities in opioid-involved overdoses and deaths.

**Methods:**

We conducted an online survey and interviews of directors of OTPs located near RCCs serving Black communities (operationally defined as located in a ZIP code where ≥25% of residents are Black, as per US Census data). For each such RCC (*n* = 47 nationwide), we used the SAMHSA Treatment Locator to identify and record data (e.g., types of opioid treatment, treatment approaches, in-house recovery support services) about the three nearest OTPs. The survey asked about the OTP’s referral practices to mutual help organizations (MHOs) and recovery support services, knowledge of and interactions with the nearby RCC, and attitudes toward referral to RCCs, including potential barriers to referral. Interviews discussed barriers and potential solutions.

**Results:**

Fifteen OTPs completed surveys (32% of targeted locations), and five directors completed interviews. OTPs participating in the survey were comparable to non-participating OTPs on Locator-reported variables. OTPs provided referral to 12-step MHOs (100%); fewer (80%) were familiar with RCCs, provided referral to RCCs (67%), or knew the nearby RCC (40%). OTP directors (100%) reported that routine referral from the OTP to RCCs makes sense and is valuable. Most common barriers were lack of knowledge of RCCs, worries that RCCs may not be supportive of MOUD use, and lack of personnel to build and maintain connections with RCCs.

**Conclusion:**

Efforts are needed to increase knowledge about RCCs among OTP leadership and staff. Needed knowledge includes general knowledge (i.e., RCCs are welcoming toward MOUDs; RCCs offer complementing support) and logistical information (e.g., RCC opening hours, transportation, that services are free).

## Introduction

The opioid epidemic is a major public health problem worldwide (1). Globally, more than 60 million people struggle with opioids (2). By a wide margin, most impacted are the United States (3), where the national overdose death rate remains unacceptably high, claiming over 107,000 lives in 2022 alone (4). It has more than doubled since 2015. Primary drivers of this death rate are synthetic opioids other than methadone (primarily fentanyl) and non-opioids incorporated into opioids (primarily xylazine). The COVID-19 pandemic has substantially worsened the overdose crisis (5, 6). Black Americans face significant disparities in overdoses and overdose deaths. Prior to the onset of the COVID-19 pandemic (1999–2018), opioid overdose death rates were increasing most rapidly among non-Hispanic Black men and women (7). The COVID-19 pandemic has further widened health disparities, with early data suggesting that the opioid-related overdose mortality rate (per 100,000) among Black Americans was higher than the overdose mortality rate among White Americans for the first time since 1999 (8).

In the US, the current gold-standard treatment for opioid use disorder (OUD) is long-term treatment using medications for opioid use disorder (MOUDs; i.e., methadone, buprenorphine, and naltrexone) that are approved by the US Food and Drug Administration. This practice is similar to that in other countries of comparable social, economic, health, and educational status (9). Methadone is the most frequently used MOUD in the US, used by 80% of MOUD-receiving patients, followed by buprenorphine (17%), and naltrexone (3%) (10). Current clinical practice guidelines advise that longer duration of MOUD use results in better outcomes. For treatment with methadone, 12 months is considered the minimum effective treatment length (11). These guidelines contrast starkly with patients’ perceptions and desires. Many patients define treatment success, in part, as discontinuation of MOUD, particularly the discontinuation of methadone (a synthetic opioid agonist) and to a lesser extent the discontinuation of buprenorphine (a partial opioid agonist) and naloxone (an opioid antagonist) (12). Early discontinuation of MOUD represents a critical barrier to the effectiveness of MOUDs (13). For methadone, discontinuation rates range between 46–66% during the first year, thus failing to reach the minimum effective treatment length (14–16). Approaches are needed that support persons initiating MOUD treatment to stay engaged in medication assisted recovery.

The primary avenue of accessing MOUDs in the US is through opioid treatment programs (OTPs) (17). In other countries, similar outpatient programs are often called ‘opioid substitution treatment programs’. Notably, such treatment programs are not utilized globally. For example, there appear to be no opioid substitution centers in the entire Latin American region (9). In the US, OTPs are highly structured outpatient clinics that provide medications alongside psychosocial and ancillary services. They must be certified by the Substance Abuse and Mental Health Services Administration (SAMHSA), a branch of the U. S. Department of Health and Human Services, and accredited by an independent, SAMHSA-approved accrediting body (18). The goal of providing support alongside MOUDs is to help people seeking recovery from OUD develop alternative behaviors and social interaction patterns (19). This approach is based on mounting evidence that demonstrates that MOUDs are very effective, but alone cannot serve as the cure for OUD, which is nowadays conceptualized as a chronic, relapsing disorder (20).

In recent years, peer recovery support services (PRSS) have emerged (21, 22). PRSS provide long-term support to people as they seek and navigate recovery from substance use disorder (SUD). These services are delivered by “peers,” who are people with lived experience of recovery from SUD. They provide services in a large variety of settings, including treatment settings and peer-led spaces, such as recovery community centers, recovery homes, recovery collegiate programs, and recovery high schools (22, 23). Their goal is to increase ‘recovery capital’, a construct introduced more than two decades ago by Granfield and Cloud (24), and currently understood to refer to the ‘resources and capacities that enable growth and human flourishing’ (25). Thus, in line with the multi-faceted nature of recovery (26), PRSS provide multi-faceted support. These services have emerged in several countries across the globe, including Canda, the United Kingdom (22), Ireland (27), Belgium (28) and Singapore (29), though the preponderance of the research on PRSS is based on practices in the US and Canada (22). Within the US, SAMHSA conceptualizes these services as addressing emotional, informational, instrumental, and/or affiliational needs (30).

Within the US, federal guidelines and legislation exist that encourage MOUD providers to work cohesively with PRSS (19, 31). Most recently, federal guidelines have included specific reference to recovery community centers (RCCs) in their guidance to connect patients with PRSS (32). RCCs are peer-driven, peer-run and peer-led brick and mortar places open to the community that provide PRSS, including providing assistance with basic needs and social services, provide access to technology, host a myriad of mutual help groups, and provide space, guidance and community for engaging in health behaviors and substance-free recreational and social activities. RCCs emerged during the new recovery advocacy movement, which occurred in the late 1990s in the US in reaction to pervasive re-stigmatization and criminalization of substance use problems alongside cultural pessimism about the prospects of long-term addiction recovery (33). It was a grass-roots effort, led by recovery community organizations across the US, and aided by SAMHSA’s Center for Substance Abuse Treatment’s (CSAT) Recovery Community Support Program (RCSP). Similar models have emerged in other countries. In the United Kingdom, the development of lived experience recovery organizations (LEROs) is promoted (22); in Singapore, in 2000, the Community Action for the Rehabilitation of Ex-Offenders (CARE) Network was set up, which by now works with over 100 diverse community partners, including schools and religious groups (29). In other countries, such as Belgium and Ireland (27), such organizations do not appear to exist (28). Empirical data to date have focused on RCCs in the US.

Current estimates suggest that approximately 200 RCCs are in operation across the US, funded primarily by state, local government and federal funding, with support from philanthropy (34). Regulatory standards for RCCs are scarce. In terms of facility accreditation, RCCs may voluntarily seek accreditation from the Council on Accreditation of Peer Recovery Support Services (CAPRSS), the only accrediting body in the US specifically for RCOs and other programs offering addiction PRSS (35). RCCs might also potentially seek accreditation via less recovery-focused bodies, such as the Commission on Accreditation of Rehabilitation Facilities (CARF), an independent, nonprofit accreditor of health and human services. Of the RCCs that exist today, it is not clear how many of them have accreditation and from what entity. In terms of staff credentialing, only recently have national model standards emerged. The “National Model Standards for Peer Support Certification” was created in response to the flourishing peer recovery support worker workforce, which led to the implementation of state-endorsed or state-run peer certification programs across 49 out of 50 states (36). In terms of billing, PRSS are billable in a majority of US states (37), yet it is unclear to what degree RCCs bill for the PRSS they deliver, as fee-for-service reimbursement is not in line with the kind of flexibility, responsiveness, and holistic approach that recovery-oriented organizations seek to provide for their participants (38).

Current US federal guidance urges OTP to work with RCCs. In 2023, as part of their Treatment Improvement Protocol (TIP) Series, SAMHSA published TIP 64, “Incorporating Peer Support into Substance Use Disorder Treatment Services,” which states: “Any setting that offers care and support for individuals who have problematic substance use should also offer or arrange for PSS [peer support services]. Integrating the peer position into SUD treatment programs should supplement PSS that are offered by recovery community organizations (RCOs) and recovery community centers (RCCs)—not replace them.” This clear and strong language underlines the importance of cohesive collaboration between OTPs and RCCs.

Currently, it is not clear to what extent OTP directors are aware of RCCs. Not every OTP has an RCC nearby. At present, there are more than 1,600 OTPs in the U. S. (17), compared to approximately 200 RCCs nationwide (34). A large number of OTPs, however, do have an RCC nearby, and this number is likely to increase, as PRSS in general and RCCs in particular are undergoing an explosive growth phase in terms of funding and proliferation (21). Close collaboration between OTPs and RCCs is needed to work toward lowering the national overdose death rate, especially in Black communities, as Black Americans face significant disparities with respect to opioid-involved overdose mortality rates (7, 8). Recent findings highlight that overdose deaths are predicted to increase significantly among Black men in their 30s and 40s (39), and that Black men in urban communities are particularly at risk (40). These findings underscore the urgency of improving addiction recovery support in Black communities. Supporting close collaboration between OTPs and RCCs serving Black communities is an important step toward that goal.

Notably, unlike several other peer-led settings, for which concerns have been raised regarding the supportiveness of these settings for using MOUDs (41, 42), data from a national survey of RCCs has highlighted the welcoming attitude RCCs have toward the use of MOUDs (34). Not only are RCCs welcoming toward people taking MOUDs, many RCCs have close working relationships with clinics that provide MOUDs, and would welcome closer collaborations with such clinics (34). Thus, it is important to understand to what extent OTPs are already providing their patients with linkage to RCCs, and how linkage between OTPs and RCCs could be improved.

To address this knowledge gap, we conducted an online survey and interview study with directors of OTPs, which were located near RCCs serving Black communities. This study was part of a larger NIDA-funded project that seeks to build toward a large-scale randomized controlled trial to test the effectiveness of proactively engaging people who take MOUDs with RCCs. Our goals were (1) to determine if OTPs directors were aware of RCCs in general (2), if they provided their patients with referrals to RCCs, including the specific RCC near them, and (3) if barriers existed to providing such referrals, and how such barriers could be overcome.

## Methods

### Participants

Participants were OTP directors and front desk staff of OTPs located near RCCs serving Black communities. We focused on this target group, because survey completion required knowledge about the overall patient demographics of the clinic and about the OTP’s current referral practices. The nature of our ‘ask’ was spelled out in the email inviting OTP directors to participate in this survey, where our team included that it may be helpful to consult others from the OTP to complete the survey about this OTP. Only one survey could be submitted per OTP.

The RCCs were identified using data from a previous study, in which a nationwide survey of RCCs was conducted (34). For each of the 198 identified RCCs, we determined the racial composition of the ZIP code in which the RCC was located. We operationally defined an RCC as serving a Black community, if at least 25% of the residents in their ZIP code were Black, which is approximately double the national prevalence of Black people in the U. S. according to the 2020 U. S. census (~12.4%) (43). This was an arbitrary cut-off; the intent was to identify areas in which the proportion of Black residents was much higher than in other areas. Thus, an RCC operating in such an area would be more likely to engage Black participants in their activities. Of the 198 RCCs nationwide, 47 RCCs met this criterion.

Eligibility criteria were (a) 18 + years of age; (b) employed by the MOUD-providing clinic as either a director or front-desk staff member; and (c) willingness and ability to engage in study procedures (i.e., online survey; optional interview).

### Procedure

For the 47 identified RCCs, we used the SAMHSA treatment locator (https://findtreatment.gov/locator) to identify nearby OTPs. To this end, we selected “substance use” under the header “filter,” “SAMHSA certification for opioid treatment program (OTP)” under the header “License/Certification/Accreditation,” and “Federally Certified Opioid Treatment Program” under the header “Type of Opioid Treatment.” We then printed the results for OTPs identified this way within a 50-mile radius of each of the 47 RCCs.

For each of the 47 RCCs, study staff then called the nearest OTP and asked to speak with the OTP director. Study staff also requested an email address to which they could send the formal invitation to participate in the online survey. This email contained the study fact sheet, and a unique survey link for each OTP. Study staff followed up with OTPs at least four times after the initial phone call (by phone and email, if available), with at least three days between each follow-up attempt. If the OTP did not complete the survey after these contact attempts, study staff contacted the next closest OTP. This process was repeated until we had reached out to three OTPs per targeted location, or all OTPs within 50 miles of the targeted RCC, if this number was less than three.

At the end of the survey, participants were invited to also complete a Zoom interview with study staff to gain deeper insight into the topics discussed in this survey. In particular, we want to know more about how clinics such as yours can network with recovery community centers, and what obstacles may stand in the way of closer collaboration.” OTP directors were offered $50 to complete the survey, and $50 to complete the interview. The survey remuneration is in line with what we offered to RCC directors in a survey on RCCs (33). The remuneration amount is relatively high because survey responses are knowledge-based questions about the OTP patient population and practices. Answering these questions may require consultation of internal reports or drawing on insight from multiple staff members that the survey respondent would have to consult in answering the questions. The interview remuneration amount was chosen to sufficiently incentivize OTP directors, whose time is very limited. All study procedures were approved by the institutional review board of the Mass General Brigham (MGB) not-for-profit, integrated health care system.

### Measures

We collected data from three sources (1): information displayed on the SAMHSA Treatment Locator about each OTP (2), survey data, and (3) interview data.

#### SAMHSA treatment locator data

For each OTP we reached out to, we recorded information from the SAMHSA Treatment Locator on five variables (see Supplementary Table 1 for details): types of opioid treatment (the SAMHSA Treatment Locator lists 13 types), pharmacotherapies (16 types), treatment approaches (12 types), in-house recovery support services (7 types), and community outreach (“outreach to persons in the community” listed / not listed under “Assessment/Pre-treatment”).

#### Survey

The survey was administered via REDCap, a secure, web-based HIPAA-compliant data capture system (44), and was structured into four sections (1): “Recovery support outside of the clinic,” (2) “The RCC near you,” (3) “Information about your clinic,” and (4) “Information about you.” In “Recovery support outside of the clinic” (section 1), we asked if participants were familiar with specific mutual help organizations, and if they provide referral to them. The same answer choices were used for all mutual help organization options, using the check-all-that-apply format: Alcoholics Anonymous (AA), Alcoholics Anonymous (AA), Narcotics Anonymous (NA), Cocaine Anonymous (CA), Celebrate Recovery, SMART Recovery, Women for Sobriety, and other. Using the same format, we then asked about recovery support services, using these answer choices: recovery coaching, recovery housing / sober living environments, faith-based recovery services, recovery high schools / Collegiate programs, recovery community centers, recovery cafes, and online recovery communities. In choosing these response options, we built on the US National Recovery Study, which used these same categories for mutual help vs. peer recovery support services (45). We added an emerging option for mutual help organizations (i.e., Celebrate Recovery) (46).

We then provided some detailed knowledge about RCCs by asking: “Each RCC is unique, but they are more alike than different. A recent nationwide survey of RCCs described the services they offer. Which services do you think most (>70%) RCCs offer?” A check-all-that apply list listed the same services and activities as asked in our prior research (34), including recovery coaching; peer-facilitated recovery support groups; employment, education, housing and legal assistance, financial services; health insurance education; mental health support recreational / social activities; opportunity to volunteer / “give back”; childcare services; family support services; NARCAN training and/or distribution; recovery advocacy outreach and opportunities; technology/internet access; basic needs assistance (e.g., access to food, clothing, transportation), expressive arts (e.g., arts/craft groups, music, poetry), health, exercise, and nutrition programs. This survey item builds on our nationwide survey of RCCs in that it uses the same list of services as assessed in that study. The impetus to ask this question stems from our community-engaged research process. Namely, our group leads an online monthly seminar series (since 2020) that brings together people from diverse key communities relevant to advancing the science on RCCs (e.g., RCC directors, RCC staff, RCC participants, RCC funders, clinicians, researchers, community members, people with lived experience, etc.) (47). In this setting, the lack of awareness by prescribers of RCCs has been highlighted repeatedly anecdotally, but lacking quantitative data. Thus, we included this question here.

We then asked a yes/no question: “MOUD-providing clinics, such as your own, and RCCs share the same goal: supporting people in recovery. They may, however, come from different perspectives to meet this need. Do you think it would make sense for clinics, such as your own, to routinely provide patients with referral to nearby RCCs for additional support?”

Next, we asked: “What kinds of concerns come to your mind (or get talked about in your clinic) when thinking about providing your patients with referrals to RCCs?” Answer choices, in the check all that apply format, were: “worry that peer-based programs discourage the use of MOUDs - will RCC staff or members be against MOUDs?,” “not knowing if RCCs will be able to help our patients - not sure what the evidence is to date,” “safety concerns for my patients - is it physically safe for them to go there?,” “information overload - we are already telling patients about too many things, this is just too much information,” “just not a good fit - RCCs may be great for some, but they are just not right for our patients,” “not knowing enough about RCCs - we just simply do not know enough about RCCs; how can we send our patients there without knowing more about them?,” “Logistical problems (e.g., funds, staff time to identify RCCs)” and “other.” The response options were derived from discussions occurring in the aforementioned seminar series (47), as well as personal experiences in having conversations about linkage to RCCs with MOUD providers (our authorship team includes RCC directors and RCC staff).

Next, we offered three write-in text boxes (1): “What do you think would be the ideal way for MOUD-providing clinics, such as your own, to connect their patients with RCCs?,” (2) “What stands in the way of making this ideal referral method possible?,” and (3) “What could be done to overcome these barriers?”

After prompting participants in this way to think about RCC services, barriers, and potential solutions, we asked the yes/no question: “Do you believe it is or would be valuable to tell your patients about RCCs?”

**Figure 1:**
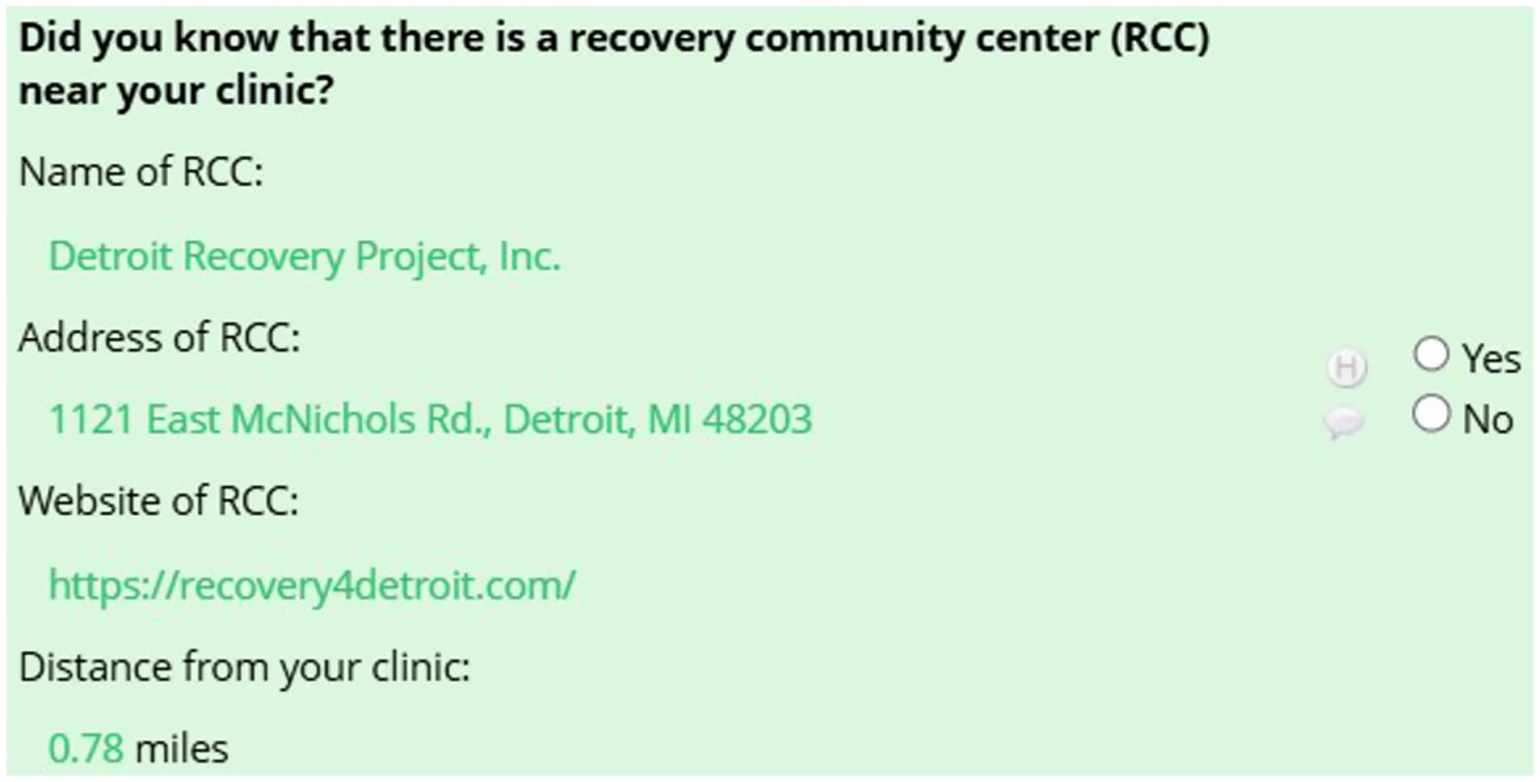
Screenshot of the survey information shared with OTP directors about their nearby RCC.

Participants then clicked “submit” to get to the next page. On this page (section 2), we asked: “Did you know that there is a recovery community center (RCC) near your clinic?” Information was piped in to show the nearby RCC’s name, address, website, and distance from the OTP (see Figure 1).

If participants clicked “yes,” they were asked questions about their interactions with this RCC: “Does your clinic have any interactions with this RCC?” “Do you tell your patients about this RCC and the services and resources it can offer them?” and “How do your patients hear about this RCC at your clinic?” Answer options are shown in the tables in the results section.

In section 3, “Information about your clinic,” we asked participants to estimate the age, gender, race, and ethnicity of their patients by providing the percentage of their patients that would best be described by specific categories (e.g., % female; see answer options in tables in the results section). We used the same response categories as in our prior research on RCC participants (34), so as to enable direct comparisons. If percentages did not add up to 100% within the four variables, an error message was displayed, which requested corrections. In this section, we also piped the SAMHSA information about whether their clinic engages in “outreach to persons in the community,” and asked them if this information was correct.

In section 4, “Information about you,” we asked about the participants’ role at the clinic, years of employment at this clinic, gender, race, ethnicity, education, and if they were in recovery themselves, and if so, for how many years.

#### Interviews

The goal of the interviews was to dive deeper into participants’ survey responses to the open-ended questions. Right before conducting the interview, study staff reviewed each participant’s survey answers, noting general background information (e.g., the size of their clinic in terms of staff and patients), awareness of RCCs, and topics raised in their open-ended responses. During the interview, study staff asked participants to revisit these three open-ended questions, which asked about ideal ways to connect with RCCs, things that stand in the way of making referrals, and ways in which these barriers could be overcome, and were asked to elaborate on their answers from the survey. The interview style was conversational, where study staff asked participants to elaborate on their original written response. Specifically, participants were prompted to elaborate on their thinking about the issues they had raised and what impact they had in the specific context of their clinic. All interviews were conducted via Zoom by one or two trained study staff (AW, CN, AF, BH) and lasted approximately 30 min. Participants received an additional $50 for completing the interview if they opted into payment. This use of triangulation between survey responses and interview questions is in line with common rapid analysis approaches for qualitative data (48). Interviews were audio-recorded and transcribed within 1–2 days of completion.

### Analysis

To test for systematic differences between the OTPs that participated in our study versus those that did not, we performed chi-square tests on the variables recorded from the SAMHSA Treatment Locator. We treated each possible answer option as a binary variable, resulting in 49 chi-square tests across the five conceptual variables. We used the Fisher’s exact test when ≥25% of the cells had expected counts less than 5. Since our statistical power was limited, we also compared participating vs. not-participating OTPs descriptively. To this end, we calculated the absolute difference in percentage points between participating vs. non-participating OTPs, and made note of variables on which this difference exceeded 20%.

To analyze the survey data, we used descriptive statistics (i.e., means with standard deviations for continuous variables or percentages and counts for categorical variables).

To analyze the interview data, we used a rapid analysis approach, in line with the emergence of these approaches in healthcare research (49). Three authors (BH, AW, CN) independently reviewed the transcripts to identify specific thoughts (barriers to referral to RCCs or solutions to overcome these barriers). Coders started by independently coding the first interview. They then compared their lists of identified thoughts and created a list of thoughts, arrived at by consensus. They then coded the next interview, flagged in each interview the presence of the already identified thoughts, and flagged new thoughts found in the new interviews. In a consensus meeting, the three coders compared their coding, and updated the list of identified thoughts by consensus. In this iterative process, they then coded interviews one-by-one, and arrived at a final list of identified barriers and solutions.

## Results

### Survey completion

In total, we reached out to 120 OTPs to obtain surveys from the 47 targeted locations. Of these, we received completed surveys from 15 OTPs (32% of targeted locations), failed to receive a survey after contacting the three closest OTPs at 60% of the targeted locations, and failed to receive a survey for all (less than 3) OTPs within a 50-mile radius of the targeted RCC at 8% of the targeted locations.

When comparing OTPs that participated in our study versus those that did not on the variables we recorded from the SAMHSA Treatment Locator (see Supplementary Table 1), only one variable had a statistically significant difference. OTPs that participated in the survey listed using Motivational Interviewing as a treatment approach less often that OTPs that did not complete a survey (80% vs. 97%, *p* = 0.03, Fisher’s Exact Test). We noted differences exceeding 20 percentage points on an additional five variables (Supplementary Table 1). For treatment approaches, OTPs that responded to survey requests more often indicated using the Matrix Model (60% vs. 33%) and 12-Step Facilitation (67% vs. 45%). For pharmacotherapies, OTPs that responded to survey requests provided the medication Lofexidine less often (7% vs. 30%). For in-house recovery support services, OTPs that responded to survey requests more often provided housing services (93% vs. 73%) and recovery coaching (53% vs. 32%).

### Staff completing survey

Surveys were completed most often by OTP directors (80%) and less frequently by staff with roles such as administrator coordinator, chief operating officer, and clinical supervisor. Survey participants had worked at their clinic for (M ± SD) 11.0 ± 10.0 years on average. Most of the survey participants were female (80%), had a master’s degree (66%), and were White (73%); four were Black (27%), and one was American Indian (7%). None were Hispanic. Two participants were in recovery themselves (13%), with an average of 21.5 ± 16.2 years in recovery.

### Description of OTPs

According to data from the SAMHSA Treatment Locator, all surveyed OTPs provided methadone, most provided buprenorphine, and many provided naltrexone (Table 1). All OTPs provided recovery support services, where the most commonly provided services were housing services (93%) and assistance with obtaining social services (87%).

**Table 1:** Description of participating MOUD-providing clinics (*n* = 15).

Variables	M/%	(SD/n)
Reported in SAMHSA treatment locator		
MOUDs provided by the clinic		
Methadone	100.0	(15)
Buprenorphine	86.7	(13)
Naltrexone	40.0	(6)
Self-reported by clinic director (or delegate)		
Number of years clinic has been in operation	23.8	(15.8)
Estimated number of people served per week	370.1	(284.9)
Number of staff		
Employed	28.2	(29.1)
Volunteer	0.5	(0.9)
Engages in outreach to community (% yes)	80.0	(12)
Demographics of patients		
Age		
Under 18 years	0.1	(0.3)
18–24 years	14.1	(13.3)
25–59 years	68.5	(16.0)
60 + years	17.3	(11.1)
Gender		
Male	62.7	(7.6)
Female	37.0	(7.7)
Other	0.3	(0.8)
Race (reported in means and SD, where each OTP estimated the percentage of patients falling into each category)		
American Indian or Alaska Native	1.0	(1.5)
Asian	0.9	(1.5)
Black	22.8	(18.3)
Native Hawaiian or Pacific Islander	0.7	(1.3)
White	68.4	(22.6)
More than one race	6.2	(7.3)
Ethnicity (% Hispanic)	15.1	(16.7)

Survey responses (Table 1) indicated at OTPs had been in operation for 23.8 ± 15.8 years and were operated by 28.2 ± 29.1 staff members, on average. Most OTPs (80%) engaged in community outreach. OTP directors estimated, on average, that their patient population consisted largely of White (68%) and Black patients (23%); they estimated that 15% of their patients were Hispanic.

### Awareness of and referral to recovery support outside of the OTP

When asked about awareness of and referral to MHOs (Table 2), all participants reported that they were familiar with and provided referral to Alcoholics Anonymous and Narcotics Anonymous. They were less familiar with other MHOs (e.g., SMART Recovery, Celebrate Recovery). Regarding recovery support services, all OTPs were familiar with recovery housing, but only 80% were familiar with RCCs; only 67% provided referral to RCCs. In general, referral to recovery support services was less common than familiarity with them.

**Table 2:** Awareness of and referral to recovery support outside of the clinic (*n* = 15).

Variables	Is familiar with		provides referrral to	
	%	(n)	%	(n)
Mutual help organizations				
Alcoholics anonymous (AA)	100.0	(15)	100.0	(15)
Narcotics anonymous (NA)	100.0	(15)	100.0	(15)
Cocaine anonymous (CA)	66.7	(10)	60.0	(9)
Celebrate recovery	66.7	(10)	66.7	(10)
SMART recovery	73.3	(11)	60.0	(9)
Women for sobriety	40.0	(6)	26.7	(4)
LifeRing	6.7	(1)		
Other	13.3	(2)	13.3	(2)
Recovery support services				
Recovery housing/Sober living environments	100.0	(15)	93.3	(14)
Faith-based recovery services	86.7	(13)	73.3	(11)
Recovery coaching	80.0	(12)	80.0	(12)
Recovery community centers	80.0	(12)	66.7	(10)
Online recovery communities	66.7	(10)	60.0	(9)
Recovery high schools/Collegiate programs	26.7	(4)	13.3	(2)
Recovery cafes	13.3	(2)	0.0	(0)

### Interactions with nearby RCC

On average, the nearby RCC was located 8.9 ± 10.1 miles from the surveyed OTP. Only 40% of the OTP directors and front desk staff surveyed knew that this RCC existed (Table 3). For those OTP directors and front desk staff that were familiar with their nearby RCC (*n* = 6), the most common type of interaction was OTP staff visiting the RCC (67% of OTPs engaged in this activity). In many cases, RCC staff had proactively introduced themselves to OTP staff (50%), and OTPs and RCCs stayed connected via emails and other online activities (50%). Regular interactions between OTPs and RCCs were rare (33%, 2/6).

**Table 3:** Clinic’s relationship with specific RCC near them.

Variables	M/%	(SD/n)
Logistics		
Distance from clinic to RCC *(in miles)*	8.9	(10.1)
Did you know about this RCC? (% Yes)	40.0	(6)
Of clinics which knew about their local RCC		
Type of interactions		
Members of our clinic staff have visited this RCC	66.7	(4)
RCC staff have introduced themselves to our clinic staff	50.0	(3)
We stay connected with this RCC via email/online	50.0	(3)
Members of our clinic staff regularly interact with RCC staff	33.3	(2)
Other	16.7	(1)
Percentage of patients who hear from clinic about this RCC		
0% of patients	0.0	(0)
1–15% of our patients	33.3	(2)
16–84% of patients	50.0	(3)
85 + % of patients	16.7	(1)
How patients hear from clinic about this RCC		
Clinical staff mention this RCC to patients	100.0	(6)
We employ recovery coaches who tell our patients about this RCC	50.0	(3)
There are flyers of this RCC in our reception area	33.3	(2)
Referral to this RCC is part of our regular end of visit notes	16.7	(1)

Only staff at one OTP (17% (1/6) of OTPs where directors or front desk staff knew the nearby RCC existed) told their patients about the RCC as part of standard of care (i.e., told 85% or more of their patients about the RCC). More commonly, OTP staff told a subset of their patients (16–84% of patients) about the nearby RCC (50%, 3/6), with staff at some OTPs (33%, 2/6) telling a very small subset of their patients about RCCs (1–15% of patients).

If patients heard about the nearby RCC from staff at their OTP, they heard about them from clinical staff [happened in 100% (6/6) of OTPs], or recovery coaches employed by the OTP [50% (3/6) of OTPs]. Written information was rarely provided: 33% (2/6) of OTPs had flyers about the RCC in their waiting area, and one OTP (17%) included written information about the nearby RCC as part of end-of-visit materials.

### Thoughts on linkage from OTP to RCC

All OTP directors and front desk staff surveyed (*n* = 15) agreed that it was valuable to tell their patients about RCCs and indicated that it made sense to provide routine referral to RCCs.

The most commonly reported barrier to providing referral to RCCs was lack of knowledge about RCCs [47% (7/15) of surveyed OTPs]. Lack of scientific knowledge about the effectiveness of RCCs in supporting patients was a specific barrier in this regard (33%, 5/15). Lack of knowledge about logistical issues also represented a barrier (33%, 5/15). A considerable number of OTPs also expressed concern about RCC potentially discouraging the use of MOUDs (40%, 6/15), which would undermine their treatment plans. Concerns about patient safety (20%, 3/15) and fit (20%, 3/15) were less common (Table 4).

**Table 4:** Thoughts on linkage from clinic to RCC.

Variables	M/%	(SD/n)
Overall impressions		
Valuable to tell your patients about RCCs (% yes)	100.0	(15)
Routine referral from clinics to RCCs makes sense (% yes)	100.0	(15)
Barriers that come to mind/are discussed by clinic staff		
Not knowing enough about RCCs - we just simply do not know enough about RCCs; how can we send our patients there without knowing more about them?	46.7	(7)
Worry that peer-based programs discourage the use of MOUDs - will RCC staff or members be against MOUDs?	40.0	(6)
Not knowing if RCCs will be able to help our patients - not sure what the evidence is to date	33.3	(5)
Logistical problems (e.g., funds, staff time to identify RCCs)	33.3	(5)
Safety concerns for my patients - is it physically safe for them to go there?	20.0	(3)
Information overload - we are already telling patients about too many things, this is just too much information	20.0	(3)
Just not a good fit - RCCs may be great for some, but they are just not right for our patients	13.3	(2)
Other	6.7	(1)

### Further insights from interviews

In interviews (*n* = 5), OTP directors further elaborated on barriers and solutions. Regarding lack of knowledge, OTP directors explained that they needed to know specific information about the kinds of services the nearby RCC provides, and if patients would be asked to pay for these services, or which of the services they would be billed for. For example, one OTP director noted that they compile lists of various resources, but then problems arise: “…and it turns out to not be [accurate]…But if we could have a definitive, this is where you go for this, this is where you go for this, and we are the best people for this…It takes a village …” Another OTP director stated, “We have at the clinic different places that we outsource and resource people to. And being able to know that you guys [RCCs] are there and the services you provide, you’ll be getting referrals daily.”

OTP directors also felt that effective referral to RCCs would need to include information about the opening hours of the RCC, and transportation opportunities that might exist to help patients get to the RCC. Transportation was noted several times as being critical. For example, one OTP director noted: “…one of the biggest challenges we have is transportation. It’s certainly not their responsibility [the patients], but we have a lot of patients who are taking the bus… and do not have the opportunity to get to a particular place as conveniently as you would like them to.” RCCs need to be nearby, and transportation accessible to enable patients to make the trip.

OTP directors also noted that it would be particularly helpful for RCC to highlight if they offer any kind of support in term of childcare, as that would greatly facilitate patients’ ability to participate in RCC activities. “I mean, providing childcare could be a big plus, at least for some people…definitely childcare and programs for new mothers kind of or even just mothers. Families, family care.”

More generally regarding knowledge gaps, OTP directors noted that emerging terms and terminology can be frustrating and confusing (e.g., RCCs vs. RCOs). One participant said, “I think the terminology is what blocks us from even knowing that we are already connecting individuals to these communities. So yeah, knowing exactly what the community is and what it stands for.” Another participant expanded upon this issue of inconsistent terminology and highlighted the need for more education on what RCC’s provide for services: “Different clinicians probably have different levels of understanding [of RCCs], and that’s why a unified training or something like that probably would be helpful to get everyone on the same page and everyone at the same kind of knowledge base.”

In terms of interacting cohesively with RCCs, OTP directors expressed that OTPs are very busy places, and staff are already stretched very thin. Thus, it is difficult to engage in the types of activities that would foster close collaboration between their OTP and nearby RCCs. “So, the more support staff that we have is a better thing. We’re about to add an intake coordinator and so on and so forth, someone who can help with referrals for other services if we need a higher level of care or something like that.” They suggested that additional funding to protect staff time to engage in these activities would go a long way to help support better linkage between OTPs and RCCs.

In general, OTP directors expressed that it would be tremendously useful to bring together OTPs, RCCs, and other organizations seeking to support people in recovery from OUD in a setting where they could connect better: “There needs to be a table for us to come to.” They suggested that perhaps such a larger coming together could result in the creation of a helpline that connects people to a variety of resources, including OTPs and RCCs, or a centralized ‘intake’ unit.

Several ideas emerged for creating and maintaining connections between RCCs and OTPs. Regarding the initial connection, OTP directors offered that RCCs calling them first would be a great first step, which should be followed by an in-person meeting. “A training would probably be the best or most effective way to educate the [OTP] staff [on RCCs]. Reading material, Zooms, things like that. But if we have someone coming in person…to be there to answer questions and respond to any questions or comments, that probably would be the most effective way to bring staff up to date with what’s really going on with this [RCCs].” Flyers and pamphlets describing the services the RCC provides would also be helpful.

Once a connection is established between the OTP and RCC, several practices could help maintain and develop this connection over time. “If it’s just a matter of them dropping off resources, then I think that would be sufficient. If it’s something where they have new information, or there’s been any changes, or there’s been new developments, then probably it would be good for them to reach out and actually be able to contact me or another staff member and be able to discuss that.” Soft hand-offs (OTPs letting RCCs know a patient will drop by, and RCC staff providing follow-up on how that person connected at the RCC), dropping off updated written information about the RCC, and emailing and calling about major updates (e.g., new services, new location, new hours) were the most commonly mentioned methods. OTP directors in particular noted that maintaining an informative and easy to navigate website would help.

## Discussion

In this study, we conducted surveys and interviews with directors of OTPs to gain insights into the extent to which OTPs are aware of RCCs and are connecting their patients with RCCs. Providing such connections is explicitly encouraged in recent federal guidelines for OTPs (32). Our study focused on OTPs near RCCs serving Black communities, as Black Americans face significant disparities in opioid-involved overdose mortality rates (7, 8). In this regard, the first important contextual finding is that only 23% of patients in the surveyed OTPs were Black, despite being located near RCCs located in ZIP codes where at least 25% of the residents were Black. By contrast, the percentage of Black individuals served by RCCs in such ZIP codes is 45% (50). This suggests that RCCs are better able to engage Black Americans in their services, and thus maybe a particularly supportive environment for Black Americans taking MOUDs.

Our results suggest that a shockingly low percentage of OTPs surveyed (40%, 6/15) were aware of RCCs that are located nearby (on average, 10 miles away). The concept of RCCs was more familiar to OTP directors than knowledge of the specific RCC down the street. Similarly striking was the hesitancy of surveyed OTP directors and front desk staff to provide patients with referral to RCCs. Such referrals were seen as universally acceptable and were provided for 12-step MHOs, such as Alcoholics Anonymous and Narcotics Anonymous. By contrast, when asked about RCCs, not all OTP directors were familiar with RCCs, and even fewer reported that their OTP provided referral to them.

Discussion of barriers to providing referrals provided important insights. The number one barrier to providing their patients with referrals to RCCs was lack of knowledge. This barrier sets RCCs apart from 12-step MHOs, which have existed for over a century, have been depicted in movies since the 1950s (51), have more than a million members in the US alone (52), and thus are a readily familiar concept to clinicians and patients alike. RCCs, on the other hand, are a relatively new concept. RCCs grew out of the recovery advocacy movement in the late 1990s (33). To date, to the best of our knowledge, only ~200 RCCs exist nationwide (34). Moreover, while some RCCs have been in operation for several decades, including the Wolfe Street Foundation (50 + years), and the RCCs participating on this research project, the Northern Ohio Recovery Association (20 + years), the Detroit Recovery Project (10 + years) and the PILLARS (7 + years), more than half of the RCCs nationwide have been in operation for less than 5 years (34). Thus, in general, there is less familiarity with RCCs.

Perplexingly, however, OTP directors noted worries that RCCs may discourage their patients from taking MOUDs as a barrier to providing referral to RCCs. This worry is contrary to recent evidence that showcases that RCCs provide a welcoming environment for people using MOUDs, and that RCC staff and participants openly discuss and facilitate MOUD use (34). Given the recency of this finding, it is not surprising that OTP directors may not be familiar with it. What is perplexing, however, is that this same worry does not appear to impede referrals to Narcotics Anonymous, where taking MOUDs can hold people back from fully participating (53). Perhaps RCCs are still being held to a more skeptical standard, simply because RCCs are still new to the field (54).

In interviews, OTP directors provided vivid insights and concrete suggestions for building toward better linkage between OTPs and RCCs. This feedback was rich and helpful. It also conveyed a welcoming stance to wanting to learn more about RCCs and engage with them. Given the many pressures, complexities, and stigma (the “not-in-my-backyard” problem) OTPs need to navigate daily, this welcoming stance is particularly encouraging. On the broad stroke, increasing knowledge and awareness appears to be the most important next step. The specific suggestions provided by OTP directors provide practical tips and steps that can serve as important starting points for working toward a more cohesive collaboration.

To increase collaboration between OTPs and RCCs, the 12-Step Facilitation treatment approach may be a suitable starting place. 12-Step Facilitation interventions seek to connect people with problematic substance use to 12-Step mutual-help organizations (e.g., Alcoholics Anonymous) by encouraging meeting attendance and providing warm hand-offs (55). This type of intervention is widely used, including by 67% of the OTPs participating in this study (see Supplementary Table 1). A recent Cochrane review found 12-Step Facilitation to be effective, reporting that it performed on par with established active comparison treatments (e.g., cognitive behavioral therapy) (56). Thus, manualized treatment models exist that link OTP patients to community resources. In linking OTP patients to RCCs, however, community-specific tailoring would be essential. While the overall service model of RCCs is shared across RCCs, each RCC is unique in many ways (e.g., staff size, look-and-feel, services, social events, etc.; for more vivid insight, consider watching the 2–8 min videos of 30 + RCCs featured in our seminar series) (57). Thus, successful linkage from OTPs to RCCs would likely require the provision of very specific information about the nearby RCC (e.g., opening hours, transportation, how services are provided, costs, if any). Such linkage would likely be supported by OTP staff knowing RCC staff, so that the OTP can stay up to date on RCC events, and so OTP staff can readily answer their patient’s questions about the RCC.

We would like to add an exploratory thought, speaking well beyond the data provided by this study, but inspired by it. In interviews, OTP directors alluded in their comments to the larger network space, or perhaps, lack thereof, that OTPs and RCCs exist in. Recently a new network model has emerged, the Hub-and-Spoke model, in which OTPs act as ‘hubs’ (58). This is a clinical model, developed in Vermont to address limitations in access to MOUDs (58), which has since been replicated in other states (59, 60). In the Hub-and-Spoke model, the hub is a central clinical organizational unit that manages patient intake, provides coordinating care, and coordinates with and refers to additional or follow-up service providers. The spokes are clinical or non-clinical service providers that provide additional services or take over care once people with OUD have stabilized care. In considering the many constraints in staffing and time OTPs are already subject to, one wonders if perhaps OTPs are not the best entities to serve as network ‘hubs’. Perhaps RCCs would be more suited to serve as ‘hubs’; not as clinical hubs, but as hubs of recovery networks, in which OTPs are part of but not the central organizing hub, in line with the recently proposed RCC-OBOT model (61). RCCs already act as a hub of PRSS in communities, with many RCCs serving as the largest providers of PRSS, largest employers of peer recovery support workers (PRSWs), and largest trainers of future PRSWs in a particular community. If closer collaboration between RCCs, OTPs and other SUD treatment settings could be achieved, perhaps RCCs would be ideally suited to facilitate networking and interconnectivity, given their expertise in community engagement, advocacy, and outreach.

### Limitations

A key limitation of our data is the small sample size. Our focus on RCCs serving Black communities certainly contributed to this small sample size, as it confined our outreach efforts to very specific locations. We would have achieved a higher sample size if we had also considered OTPs near any RCCs, not just RCCs serving Black communities. We believe, however, that the larger problem was the low response rate. Having engaged in a survey of this nature with RCC directors, we naively expected response rates to be similarly high (62% of 198 RCC directors answered our survey in a previous study) (34). Surveying OTP directors proved to be much more challenging, however, than anticipated. Our overall response rate for targeted locations was 32%, which is on par with the response rate of a national survey of OTPs, which achieved a 32% response rate for contacted OTPs (17). In our case, however, we contacted up to three OTPs for each location, so that our overall response rate to survey invitation was much lower (i.e., 13%). It is possible that lack of familiarity with the survey topic could have led to survey non-response. Our written survey invitation specifically highlighted our intent to learn about “if and how clinics, such as yours, connect their patients with recovery support services.” We expected the term ‘recovery support services’ to be familiar to OTP directors, because federal guidelines for OTPs specifically reference the need to connect patients with recovery support services (32), and because OTPs provide data to the SAMHSA Treatment Locator (which we used to identify OTPs specifically) on recovery support services. It is possible, however, that OTP directors were less familiar with this term than we assumed. Fortunately, our data suggest that the OTPs we were able to survey were relatively representative of the OTPs we sought to survey, with only one statistically significant difference between OTPs we surveyed vs. OTPs we failed to engage in our surveys. Thus, while our sample size is small, it is likely representative. It should also be kept in mind that we only surveyed one person per OTP. While serving in a leadership position, survey and interview responses are limited to the individual perspectives of the specific study participants and may not fully reflect the whole staff of the organizations they were representing.

## Conclusion

Efforts are needed to increase knowledge about RCCs among OTP leadership and staff. Needed knowledge includes general knowledge (i.e., RCCs are welcoming spaces for MOUDs; RCCs offer complementing support) and specific logistical information (e.g., RCC opening hours, transportation, information on (lack of) costs).

## Data Availability

The raw data supporting the conclusions of this article will be made available by the authors, without undue reservation.
