# Supplementary material for: The linkage between opioid treatment programs and recovery community centers: results of a survey of OTP directors
Source: Front Public Health. 2025 Aug 7;13:1532374. doi: 10.3389/fpubh.2025.1532374 (PMC12369950; doi:10.3389/fpubh.2025.1532374)
Supplement: Supplementary file 1 [file Table_1.DOCX]

| *Table 0.* Predictors of engaging in the survey | |  |  |  |  |  |
| --- | --- | --- | --- | --- | --- | --- |
|  |  | Clinics who completed survey | | Clinics who did not complete a survey | |  |
|  |  | (n=15) | | (n=105) | |  |
|  |  | % | n | % | n | *p* |
| Type of opioid treatment | |  |  |  |  |  |
|  | Federally-certified Opioid Treatment Program | 100.0 | (15) | 100.0 | (105) | n/a |
|  | Prescribes buprenorphine | 66.7 | (10) | 56.2 | (59) | 0.58 |
|  | Buprenorphine used in Treatment | 0.0 | (0) | 0.0 | (0) | n/a |
|  | Buprenorphine maintenance | 80.0 | (12) | 81.0 | (85) | 1.00 |
|  | Buprenorphine detoxification | 40.0 | (6) | 26.7 | (28) | 0.36 |
|  | Methadone used in Treatment | 0.0 | (0) | 1.0 | (1) | 1.00 |
|  | Methadone detoxification | 40.0 | (6) | 26.7 | (28) | 0.36 |
|  | Methadone maintenance | 93.3 | (14) | 93.3 | (98) | 1.00 |
|  | Prescribes naltrexone | 40.0 | (6) | 30.5 | (32) | 0.55 |
|  | Relapse prevention with naltrexone | 26.7 | (4) | 27.6 | (29) | 1.00 |
|  | Maintenance service with medically supervised withdrawal after stabilization | 66.7 | (10) | 66.7 | (70) | 1.00 |
|  | Lofexidine or Clonidine detoxification | 0.0 | (0) | 8.6 | (9) | 0.60 |
|  | Accepts clients using MAT but prescribed elsewhere | 13.3 | (2) | 18.1 | (19) | 1.00 |
|  | Use methadone/buprenorphine for pain management or emergency dosing | 0.0 | (0) | 2.9 | (3) | 1.00 |
| Pharmacotherapies | |  |  |  |  |  |
|  | Acamprosate (Campral®) | 20.0 | (3) | 24.8 | (26) | 1.00 |
|  | Disulfiram | 6.7 | (1) | 21.0 | (22) | 0.30 |
|  | Buprenorphine with naloxone | 66.7 | (10) | 81.0 | (85) | 0.30 |
|  | Buprenorphine without naloxone | 66.7 | (10) | 64.8 | (68) | 1.00 |
|  | Buprenorphine (extended-release, injectable) | 20.0 | (3) | 21.0 | (22) | 1.00 |
|  | Methadone | 100.0 | (15) | 95.2 | (100) | 1.00 |
|  | Naltrexone (oral) | 33.3 | (5) | 30.5 | (32) | 0.77 |
|  | Naltrexone (extended-release, injectable) | 33.3 | (5) | 33.3 | (35) | 1.00 |
|  | Medications for HIV treatment | 6.7 | (1) | 16.2 | (17) | 0.46 |
|  | Medications for Hepatitis C treatment | 13.3 | (2) | 21.0 | (22) | 0.73 |
|  | Lofexidine; Clonidine | 6.7 | (1) | 29.5 | (31) | 0.07 |
|  | Medications for pre-exposure to prophylaxis | 6.7 | (1) | 7.6 | (8) | 1.00 |
|  | Medication for mental disorders | 40.0 | (6) | 38.1 | (40) | 1.00 |
|  | Nicotine replacement | 26.7 | (4) | 40.0 | (42) | 0.40 |
|  | Non-nicotine smoking/tobacco cessation | 26.7 | (4) | 31.4 | (33) | 1.00 |
|  | Buprenorphine sub-dermal implant | 0.0 | (0) | 10.5 | (11) | 0.36 |
| Treatment Approaches | |  |  |  |  |  |
|  | Anger management | 73.3 | (11) | 59.0 | (62) | 0.40 |
|  | Brief intervention | 66.7 | (10) | 68.6 | (72) | 1.00 |
|  | Cognitive behavioral therapy | 86.7 | (13) | 90.5 | (95) | 0.65 |
|  | Contingency management/motivational incentives | 40.0 | (6) | 55.2 | (58) | 0.29 |
|  | Community reinforcement plus vouchers | 26.7 | (4) | 14.3 | (15) | 0.25 |
|  | Motivational interviewing | 80.0 | (12) | 97.1 | (102) | 0.03 |
|  | Matrix Model | 60.0 | (9) | 33.3 | (35) | 0.08 |
|  | Relapse prevention | 93.3 | (14) | 95.2 | (100) | 0.56 |
|  | Substance use disorder counseling | 100.0 | (15) | 99.0 | (104) | 1.00 |
|  | Telemedicine/telehealth therapy | 73.3 | (11) | 73.3 | (77) | 1.00 |
|  | Trauma-related counseling | 73.3 | (11) | 70.5 | (74) | 1.00 |
|  | 12-step facilitation | 66.7 | (10) | 44.8 | (47) | 0.17 |
| Recovery Support Services | |  |  |  |  |  |
|  | Self-help groups | 53.3 | (8) | 44.8 | (47) | 0.59 |
|  | Housing services | 93.3 | (14) | 73.3 | (77) | 0.11 |
|  | Assistance with obtaining social services | 86.7 | (13) | 81.9 | (86) | 1.00 |
|  | Recovery coach | 53.3 | (8) | 32.4 | (34) | 0.15 |
|  | Mentoring / Peer support | 73.3 | (11) | 71.4 | (75) | 1.00 |
|  | Employment counseling or training | 66.7 | (10) | 49.5 | (52) | 0.27 |
|  | None | 0.0 | (0) | 6.7 | (7) | 0.59 |
| Does the clinic do outreach to persons in the community | | 80.0 | (12) | 70.5 | (74) | 0.55 |
